# Multiple, Segmental, Non-Syndromic Basal Cell Carcinomas—Clinical, Dermoscopic and Histopathological Features †

**DOI:** 10.3390/diagnostics15212739

**Published:** 2025-10-28

**Authors:** Martyna Sławińska, Beata Zagórska, Wojciech Biernat, Michał Sobjanek

**Affiliations:** 1 Department of Dermatology, Venereology and Allergology, Faculty of Medicine, Medical University of Gdańsk, 80-214 Gdańsk, Poland; beatazagorska@gumed.edu.pl (B.Z.);; 2 Department of Pathomorphology, Faculty of Medicine, Medical University of Gdańsk, 80-214 Gdańsk, Poland

**Keywords:** non-syndromic BCC, multiple BCC, dermoscopic features, histopathological features

## Abstract

We present a case of a 72-year-old woman with four amelanotic tumors on the left arm, without a history of skin cancer or sun exposure. Dermoscopy showed polymorphic and arborizing vessels, with some lesions displaying non-specific malignant features. Histopathology confirmed basal cell carcinoma (BCC) in all lesions. No signs of recurrence were observed during 3-year follow-up. Segmental/agminated basal cell carcinoma is a rare differential diagnosis of multiple clustered, painless pink tumors. To the best of our knowledge, this is the first report describing their dermoscopic features.

**Figure 1:**
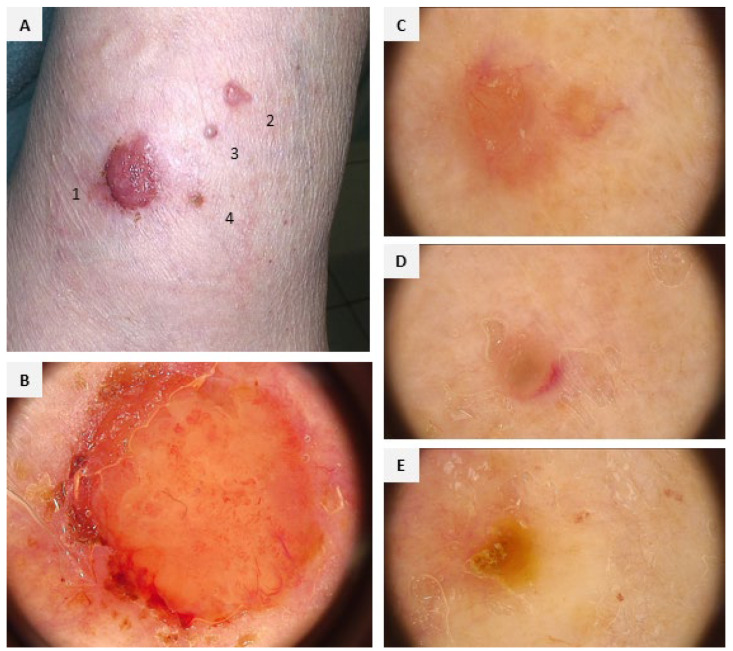
A 72-year-old woman (phototype II) presented with four amelanotic tumors on the left arm (**A**). The lesions developed on the skin area, without signs of photodamage. There was no personal or family history of skin cancer. In addition, she reported no comorbidities, excessive UV exposure, or contact with potentially cancerogenic chemicals. The largest lesion appeared around 12 months before, and the smaller ones several months prior to hospital admission. Clinical assessment did not show typical abnormalities for genetic syndromes associated with multiple BCC. Dermoscopy of the largest tumor showed polymorphic vessels over yellow-pinkish background (**B**); the smaller lesions revealed the presence of an ulceration and/or arborizing vessels (**C**–**E**). Dermoscopic features of this entity have not been previously reported, but it seems to share features observed in the BCC spectrum [1]. While the arborizing (branched) vessels in one of the tumors (**E**) could serve as a diagnostic clue, the remaining lesions presented non-characteristic patterns—yellow structureless areas corresponding to the ulceration or polymorphic vascular pattern, known as non-specific sign of malignancy.

**Figure 2:**
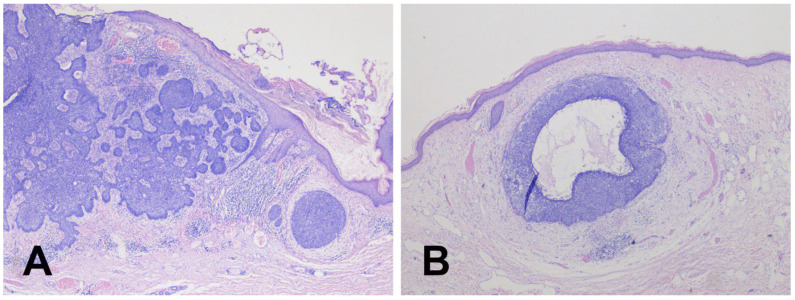
The histopathological evaluation showed typical BCC features in all lesions. Two representative images illustrate multifocal aggregation with noduloinfiltrative (**A**) and nodular superficial (**B**) growth patterns. One nodular tumor also exhibited a discrete cystic arrangement (**B**).

Basal cell carcinoma is the most common skin cancer, affecting mostly older individuals with lighter skin phototypes and a history of sun exposure. Such a presentation of multiple, segmental, non-syndromic BCCs seems to be rare, with scarce representation in the literature (Supplementary Table S1) [2,3,4,5,6,7,8,9,10,11]. None of the previously reported cases concerned lesions located exclusively on the arm. The lesions in this patient were classified as segmental due to their linear arrangement along a defined area of the left arm, suggesting a pattern that is compatible with cutaneous mosaicism. All cases included in Supplementary Table S1 similarly involved lesions restricted to one side of the body and were also classified as segmental. Although agminated basal cell carcinomas may appear as clustered lesions in photodamaged skin, the observed linear and unilateral distribution in this case supports a segmental pattern. In the literature, agminated BCC refers to localized clusters of tumors without a clear segmental or blaschkoid distribution [12,13]. This distinction is important to differentiate mosaicism-related segmental BCCs from simple agminated clustering. The etiopathogenesis of this entity remains to be unclear, but it was postulated that it may be caused by a post-zygotic mutation [4]. Although we did not perform gene or protein expression analyses in this case, rare reports have described associations of multiple non-syndromic BCCs with germline or somatic alterations in PTCH1, BAP1, or MUTYH. Given the very limited evidence, further molecular studies are needed to clarify the potential genetic basis of such cases [14,15,16].

Despite several clinicopathological subtypes, due to its distinct clinical and dermoscopic features, in most cases, the diagnosis of BCC does not pose difficulties [1,2]. Some cases of BCC may be difficult to diagnose clinically—i.e., BCC arising on the anterior surface of the lower legs presenting with polymorphic vascular pattern, totally ulcerated BCC with an unknown history of growth, or morpheaform BCC with discrete clinical and dermoscopic features. Another clinical form that is difficult to diagnose without histopathological assessment may be agminated/segmental BCC with equivocal dermoscopic features.

## Data Availability

The original contributions presented in this study are included in the article/Supplementary Materials. Further inquiries can be directed to the corresponding author.

## Supplementary Materials

The following supporting information can be downloaded at: https://www.mdpi.com/article/10.3390/diagnostics15212739/s1, Table S1. Documented Cases of Multiple, Segmental, Non-Syndromic Basal Cell Carcinoma (BCC).
