# Supplementary material for: Multiple, Segmental, Non-Syndromic Basal Cell Carcinomas—Clinical, Dermoscopic and Histopathological Features
Source: Diagnostics (Basel). 2025 Oct 28;15(21):2739. doi: 10.3390/diagnostics15212739 (PMC12608945; doi:10.3390/diagnostics15212739)
Supplement: Supplementary file 1 [file diagnostics-15-02739-s001.zip › diagnostics-3784526-supplementary.pdf]

**Supplementary Table S1.** Documented Cases of Multiple, Segmental, Non-Syndromic Basal Cell Carcinoma (BCC).

| **First author, journal, year** | **Number of cases** | **Gender/age at occurrence/age at diagnosis [years]** | **Number of lesions** | **Anatomical distribution** | **Histopathological subtype of BCC** |
|---|---|---|---|---|---|
| Moulin *et al. Ann Dermatol Venereol.* 1988 | 1 | F/40/61 | 11 | trunk (left side) | superficial |
| Bouscarat *et al. Ann Dermatol Venereol.* 1990 | 1 | M/20/92 | 30 | face, trunk, scrotum, arm, and thigh (left side only) | superficial |
| Guarneri *et al. Dermatology.* 2000 | 1 | M/30/33 | 12 | thorax (right side) | NR |
| Yoshikawa *et al. J Dermatol* 2005 | 1 | F/77/78 | 3 | abdomen (right side – 2 lesions); right lower back (1 lesion) | NR |
| *Lane et al. J Cutan Med Surg* 2005 | 1 | M/NR/56 | NR (multiple) | left side of the back, left posterior arm | NR |
| Kelly *et al.* Dermatol Surg. *2006* | 1 | F/12/13 | NR | segmental distribution over the left C4-T1 | infundibulocystic |
| Weiss *et al. J Drugs Dermatol* 2015 | 1 | M/28/61 | NR (multiple) | scalp, face, chest, inguinal fold (right side) | Nodular |
| Krajewski *et al. Acta Derm Venereol.* 2020 | 1 | F/35/55 | 31 | trunk, extremities, neck and behind the ear (left side) | 26 superficial BCC 5 nodular BCC |
| Plachouri K, et al. *BMJ Case Reports CP* 2020 | 1 | F/63/66 | 3 | lumbosacral region | NR |
| Al Aboud A, et al. *Our Dermatol Online,* 2022 | 1 | M/39/39 | 3 | forehead (right side) | infiltrative BCC |

| | | | | | |
|---|---|---|---|---|---|
| Sławińska *et al.* | 1 | F/71/72 | 4 | left arm | Superficial, nodular and noduloinfiltrative |

F – female; M – male; NR – not reported
